# Identifying and assessing the impact of key neighborhood-level determinants on geographic variation in stroke: a machine learning and multilevel modeling approach

**DOI:** 10.1186/s12889-020-09766-3

**Published:** 2020-11-07

**Authors:** Jiayi Ji, Liangyuan Hu, Bian Liu, Yan Li

**Affiliations:** 1 Department of Population Health Science and Policy, Icahn School of Medicine at Mount Sinai, 1425 Madison Avenue, New York, NY 10029 USA; 2 Institute for Health Care Delivery Science, Icahn School of Medicine at Mount Sinai, New York, NY USA; 3 Department of Obstetrics, Gynecology, and Reproductive Science, Icahn School of Medicine at Mount Sinai, New York, NY USA

**Keywords:** Neighborhood, Prevention, Bayesian machine learning, Bayesian multilevel modeling

## Abstract

**Background:**

Stroke is a chronic cardiovascular disease that puts major stresses on U.S. health and economy.

The prevalence of stroke exhibits a strong geographical pattern at the state-level, where a cluster of southern states with a substantially higher prevalence of stroke has been called the stroke belt of the nation. Despite this recognition, the extent to which key neighborhood characteristics affect stroke prevalence remains to be further clarified.

**Methods:**

We generated a new neighborhood health data set at the census tract level on nearly 27,000 tracts by pooling information from multiple data sources including the CDC’s 500 Cities Project 2017 data release. We employed a two-stage modeling approach to understand how key neighborhood-level risk factors affect the neighborhood-level stroke prevalence in each state of the US. The first stage used a state-of-the-art Bayesian machine learning algorithm to identify key neighborhood-level determinants. The second stage applied a Bayesian multilevel modeling approach to describe how these key determinants explain the variability in stroke prevalence in each state.

**Results:**

Neighborhoods with a larger proportion of older adults and non-Hispanic blacks were associated with neighborhoods with a higher prevalence of stroke. Higher median household income was linked to lower stroke prevalence. Ozone was found to be positively associated with stroke prevalence in 10 states, while negatively associated with stroke in five states. There was substantial variation in both the direction and magnitude of the associations between these four key factors with stroke prevalence across the states.

**Conclusions:**

When used in a principled variable selection framework, high-performance machine learning can identify key factors of neighborhood-level prevalence of stroke from wide-ranging information in a data-driven way. The Bayesian multilevel modeling approach provides a detailed view of the impact of key factors across the states. The identified major factors and their effect mechanisms can potentially aid policy makers in developing area-based stroke prevention strategies.

**Supplementary Information:**

The online version contains supplementary material available at 10.1186/s12889-020-09766-3.

## Background

Stroke is the leading cause of death and disability-adjusted life years worldwide, including 795,000 new stroke cases and 142,142 stroke-related deaths in the United States in 2018 [1]. There is now considerable evidence for the risk factors of stroke at the individual level. For example, stroke has been found to be correlated with modifiable risk factors, like high blood pressure, obesity and elevated cholesterol level, and unhealthy behaviors like smoking and sedentary lifestyle [2–4]. The incidence and prevalence of stroke were also shown to be markedly higher among older adults, Blacks and those with low socioeconomic status [5].

More recently, a growing number of studies reported that neighborhood context was associated with stroke incidence and mortality after stroke [6–15]. However, relatively few studies [16–20] have examined such associations when both the potential risk factors and the outcome are at the neighborhood level. Of note, three studies characterized the neighborhood-level associations for focused research questions with predetermined predictors, e.g., racial disparities in Howard G et al. [16] and Pickle LW et al. [17], and the impact of fast food restaurants in Morgenstern LB et al. [18]

Three other studies sought to identify and rank important predictors for the neighborhood-level prevalence of cardiovascular diseases at the mean level [19, 21], and for different percentiles of the response distribution [20]. A detailed summary of the related articles and methods involved and study results appear in Table 1.

**Table 1:** Literature studying associations between the neighborhood risk factors and stroke at individual-level or neighborhood-level

Outcome stroke	Paper	Methods	Results
Individual-level	Osypuk TL, Ehntholt A, Moon JR, Gilsanz P, Glymour MM. Neighborhood Differences in Post-Stroke Mortality. Circ Cardiovasc Qual Outcomes. 2017;10 (2):e002547.	Cox proportional hazard models (All individual-level variables)	Neighborhood characteristics (Race, income, age) predict post-stroke mortality, but most effects are similar for individuals without stroke.
	Menec VH, Shooshtari S, Nowicki S, Fournier S. Does the relationship between neighborhood socioeconomic status and health outcomes persist into very old age? A population-based study. J Aging Health. 2010; 22:27–47.	Multilevel logistic regressions (individual level variable and neighborhood level variable)	Relative to individuals living in the most affluent areas, those in the poorest areas had significantly higher odds of having stroke. Significant neighborhood income effects tended to be evident among individuals age 65 to 75 as well as those age 75 + .
	Brown P, Guy M, Broad J. Individual socio-economic status, community socio-economic status and stroke in new zealand: A case control study. Soc Sci Med. 2005; 61:1174–1188.	Stepwise logistic regression (all individual level variables)	Individual income and average household income are significant predictors of onset of stroke both independently and after controlling for behavioural and medical risk factors.
	Brown AF, Liang L-J, Vassar SD, Stein-Merkin S, Longstreth WT, Ovbiagele B, Yan T, Escarce JJ. Neighborhood disadvantage and ischemic stroke: The cardiovascular health study (chs). Stroke. 2011; 42:3363–3368.	Race-stratified multilevel Cox proportional hazard models (individual level variable and neighborhood level variable)	Higher risk of incident ischemic stroke was observed in the most disadvantaged neighborhoods among whites, but not among Blacks.
	Engström G, Jerntorp I, Pessah-Rasmussen H, Hedblad B, Berglund G, Janzon L. Geographic distribution of stroke incidence within an urban population: Relations to socioeconomic circumstances and prevalence of cardiovascular risk factors. Stroke. 2001; 32:1098–1103	Direct standardization with the equivalent average rate method	Socioeconomic score correlated significantly with area-specific stroke rates among men and women. Incidence of stroke was significantly associated with cardiovascular risk score for each area.
	Lisabeth L, Diez Roux A, Escobar J, Smith M, Morgenstern L. Neighborhood environment and risk of ischemic stroke: The brain attack surveillance in corpus christi (basic) project. Am J Epidemiol. 2007; 165:279–287.	Poisson regression (individual level)	In Poisson regression analyses comparing the 90th percentile of neighborhood score (median annual household income, education, occupation, housing price) with the 10th, the relative risk of stroke was 0.49 (95% confidence interval: 0.41, 0.58).
	Clark CJ, Guo H, Lunos S, Aggarwal NT, Beck T, Evans DA, Mendes de Leon C, Everson-Rose SA. Neighborhood cohesion is associated with reduced risk of stroke mortality. Stroke. 2011; 42:1212–1217	Marginal Cox proportional hazard models (individual level)	Neighborhood-level social cohesion was independently protective against stroke mortality. Research is needed to further examine observed race differences and pathways by which cohesion is health-protective.
	Brown AF, Liang L-J, Vassar SD, Merkin SS, Longstreth WT, Ovbiagele B, Yan T, Escarce JJ. Neighborhood socioeconomic disadvantage and mortality after stroke. Neurology. 2013; 80:520–527.	Multilevel Cox proportional hazard models (individual level variable and neighborhood level variable)	Living in a socioeconomically disadvantaged neighborhood is associated with higher mortality hazard at 1 year following an incident stroke.
	Aslanyan S, Weir CJ, Lees KR, Reid JL, McInnes GT. Effect of area-based deprivation on the severity, subtype, and outcome of ischemic stroke.	Stepwise linear and logistic regression (individual level)	Tackling health inequalities in stroke should focus on stroke primary prevention by tackling deprivation, including promoting changes in lifestyle.
	Gerber Y, Weston SA, Killian JM, Therneau TM, Jacobsen SJ, Roger VL: Neighborhood income and individual education: Effect on survival after myocardial infarction. Mayo Clinic Proceedings. 2008, 83 (6): 663–669. 10.4065/83.6.663.	Cox proportional hazards models	Poor neighborhood income was a powerful predictor of mortality even after controlling for a variety of potential confounding factors.
Neighborhood-level	Hu, L., Ji, J., Li, Y. et al. Quantile Regression Forests to Identify Determinants of Neighborhood Stroke Prevalence in 500 Cities in the USA: Implications for Neighborhoods with High Prevalence. J Urban Health (2020). 10.1007/s11524-020-00478-y	Quantile Regression Forests	Neighborhoods with a larger share of non-Hispanic blacks, older adults or people with insufficient sleep tended to have a higher prevalence of stroke, whereas neighborhoods with a higher socio-economic status in terms of income and education had a lower prevalence of stroke.
	Hu L, Ji J, Liu B, Li Y. Tree-Based Machine Learning to Identify and Understand Major Determinants for Stroke at the Neighborhood Level. J Am Heart Assoc. 2020; 00: e016745. 10.1161/JAHA.120.016745.	BART, Bayesian linear regression model	Of the five most important predictors identified by our method, higher prevalence of low physical activity, larger share of older adults, higher percentage of non-Hispanic blacks and higher ozone levels were associated with higher prevalence of stroke at the neighborhood level. Higher median household income was linked to lower prevalence.
	Morgenstern LB, Escobar JD, Sánchez BN, Hughes R, Zuniga BG, Garcia N, Lisabeth LD. Fast food and neighborhood stroke risk. Ann Neurol. 2009; 66:165–170.	Poisson regression and generalized estimating equations	Controlling for demographic and SES factors, there was a significant association between fast food restaurants and stroke risk in neighborhoods in this community-based study.
	Pickle LW, Mungiole M, Gillum RF: Geographic variation in stroke mortality in blacks and whites in the United States. Stroke. 1997, 28 (8): 1639–1647. 10.1161/01.STR.28.8.1639.	Multilevel regressions	Mortality rates in the Southeast also remain high, especially for Blacks.
	Howard G, Howard VJ, Katholi C, Oli MK, Huston S: Decline in US stroke mortality - An analysis of temporal patterns by sex, race, and geographic region. Stroke. 2001, 32 (10): 2213–2218. 10.1161/hs1001.096047.	Logistics regression (analyses were performed at the county level)	White men have experienced the largest decline in stroke mortality, and black men have seen the smallest. Generally, stroke mortality appears to still be slowly declining for blacks but not for whites. Geographic differences in stroke mortality are predicted to persist.
	Hu L, Liu B, Li Y. Ranking sociodemographic, health behavior, prevention, and environmental factors in predicting neighborhood cardiovascular health: A Bayesian machine learning approach. Preventive Medicine. 2020;141:106240.	BART	Neighborhood behavioral factors such as the proportions of people who are obese, do not have leisure-time physical activity, and have binge drinking emerged as top five predictors for most of the neighborhood cardiovascular health outcomes.

No studies have examined the effect mechanisms of neighborhood level risk factors on stroke prevalence while accounting for the multilevel data structure of the neighborhood health data. As a direct consequence, there is a lack of understanding about to what extent the differences in stroke prevalence in the US states can be attributed to differences in key neighborhood-level characteristics.

To fill the research gaps, our study employed a two-stage modeling approach to understand how key neighborhood characteristics affect the geographic variation in stroke prevalence. The first stage focused on identifying key determinants of stroke prevalence at the neighborhood level using a novel and precise Bayesian machine learning algorithm. The second stage used a Bayesian multilevel modeling approach to evaluate the effects of the key determinants on stroke prevalence across 49 states in the US. The investigation would provide valuable guidance for developing area-based interventions focusing on key modifiable risk factors at the neighborhood level to reduce stroke prevalence in specifically targeted areas.

## Methods

### Data source

We integrated three data sources and created a large-scale neighborhood health data [19]. Census tract was used as a proxy of neighborhood. Data on the prevalence of health outcomes, prevention, and health behavior measures were drawn from the Centers for Disease Control and Prevention (CDC)’s 500 Cities Project 2017 data release [22]. Socio-demographic measures for the selected census tracts were from the 2011–2015 American Community Survey 5-Year Estimates [23, 24]. We obtained information on environmental exposures from the Environmental Protection Agency (EPA)’s Environmental Justice Screening (EJSCREEN) database [25].

**Fig. 1:**
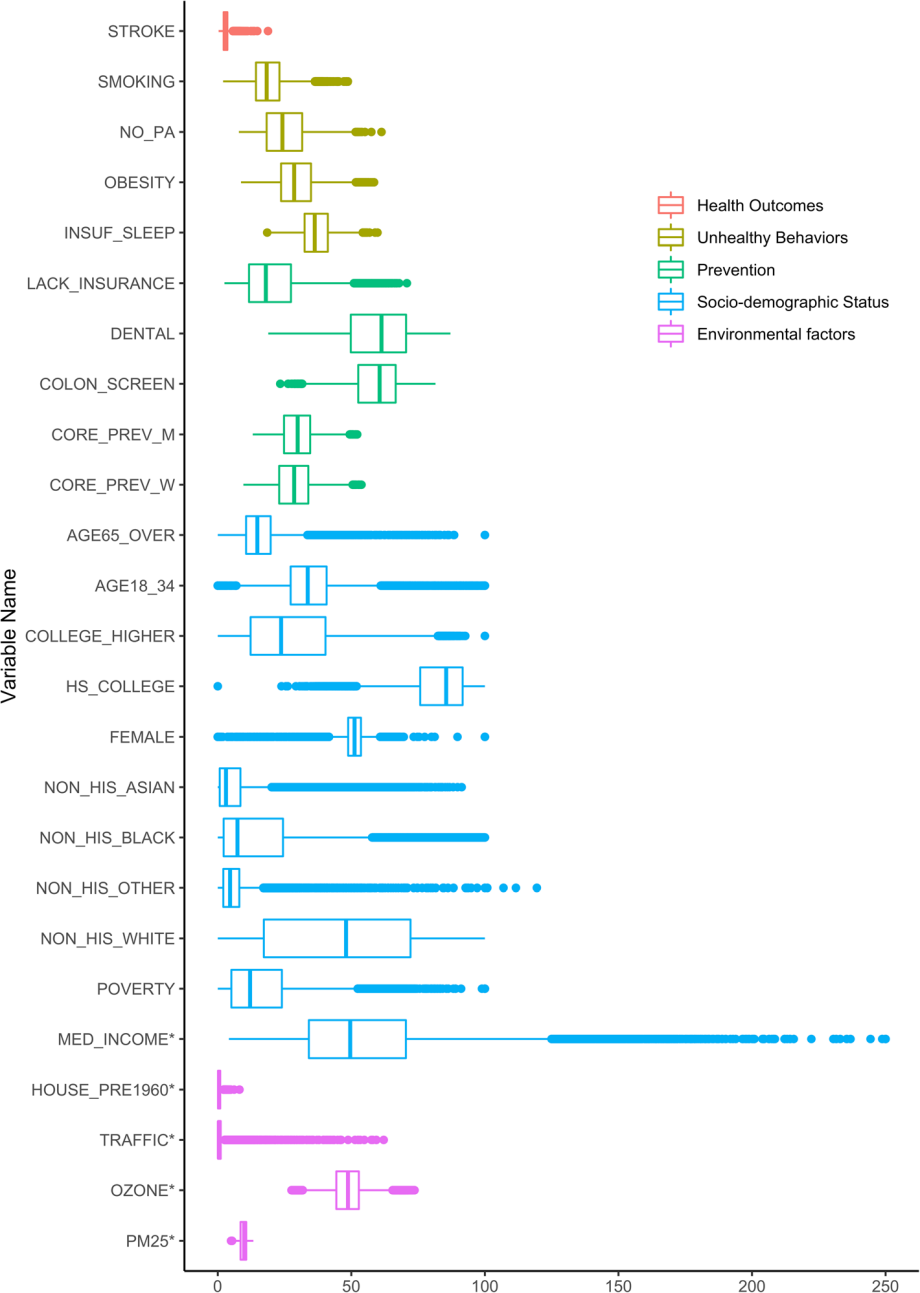
Boxplots of 24 potential neighborhood-level predictors and prevalence of stroke across 500 cities. Measures are in percentages for all variables except those marked with an asterisk, which are in absolute measurements

The main outcome of the study was stroke prevalence measured at census tract level. We included 24 potential predictors of four types, sociodemographic information, prevention measures, unhealthy behaviors, and environmental measures, which are related to cardiovascular health [20]. Detailed descriptions of the variables, their data sources and distributions are shown in Table 2 and Fig. 1. Both the outcome and the predictors were measured at the neighborhood level. After excluding missing data on key variables, our final analytical dataset included 26,697 census tracts across 49 US states. The number of census tracts in a state varies from 11 to 5368 with a median value of 307.

**Table 2:** Distribution of 24 potential neighborhood-level predictors and prevalence of stroke across 500 cities

Domain	Variable Name	Definition	Data source
Health Outcomes	STROKE	Stroke among adults aged ≥18 years	CDC 500 Cities Data
Unhealthy Behaviors	SMOKING	Current smoking among adults aged ≥18 years	CDC 500 Cities Data^a^
	NO_PA	No leisure-time physical activity among adults aged ≥18 years	
	OBESITY	Obesity among adults aged ≥18 years	
	INSUF_SLEEP	Sleeping less than 7 h among adults aged ≥18 years	
Prevention	LACK_INSURANCE	Current lack of health insurance among adults aged 18–64 years	CDC 500 Cities Data
	DENTAL	Visits to dentist or dental clinic among adults aged ≥18 years	
	COLON_SCREEN	Fecal occult blood test, sigmoidoscopy, or colonoscopy among adults aged 50–75 years	
	CORE_PREV_M	Older adults aged ≥65 years who are up to date on a core set of clinical preventive services (Men: Flu shot past year, Pneumococcal polysaccharides vaccine (PPV) shot ever, Colorectal cancer screening)	
	CORE_PREV_W	Older adults aged ≥65 years who are up to date on a core set of clinical preventive services (Women: Same as above and Mammogram past 2 years)	
Socio-demographic Status	AGE65_OVER	Population aged 65 and over	ACS^b^
	AGE18_34	Population aged between 18 and 34	
	COLLEGE_HIGHER	Bachelor’s degree or higher	
	HS_COLLEGE	High school graduate or higher	
	FEMALE	Female	
	NON_HIS_ASIAN	Not Hispanic or Latino: - Asian alone	
	NON_HIS_BLACK	Not Hispanic or Latino: - Black or African American alone	
	NON_HIS_OTHER	Not Hispanic or Latino: - Other	
	NON_HIS_WHITE	Not Hispanic or Latino: - White alone	
	POVERTY	Below poverty level; Estimate; Families	
	MED_INCOME	Median household income in the past 12 months (in thousands)	
Environmental factors	HOUSE_PRE1960	Pre-1960 housing (lead paint indicator) (in thousands)	
	TRAFFIC	Traffic proximity and volume (average number of vehicles/distance)	
	OZONE	Ozone level in air (ppb)	EPA-EJSCREEN^c^
	PM25	PM_2.5_ level in air (*μg*/*m*^3^)	

^a^ census tract level 500 Cities Data from the Centers for Disease Control and Prevention (CDC), which were modeled based on population-based survey data from the Behavioral Risk Factor Surveillance System (BRFSS).; ^b^ census tract level data from the 2011–2015 American Community Survey 5-Year Estimates provided by the Census Bureau; ^c^ To match the geospatial unit of census tract available in the other two data sources, we aggregated the census block group level environmental measures to the census tract level by taking the means for PM_2.5_ and O_3_, and the sum for the housing data, and the sum of block-group-level population weighted traffic data. PM_2.5_ concentrations are annual average of the daily ambient average, and ozone concentrations are average of daily maximum 8-h level for the summer season. Both PM_2.5_ and ozone were from a space-time downscaling fusion model based on monitoring data and modeled data. Traffic data reflect annual average daily traffic count of vehicles, i.e. count of vehicle at major roads within 500 m divided by distance in meters, and was calculated based on traffic data from the U.S. Department of Transportation. Pre-1960 housing data were based on ACS from the U.S. Census

### Statistical analysis

We identified key neighborhood-level determinants of and their impacts on the neighborhood-level stroke prevalence using a two-stage modeling approach. In the first stage, we used Bayesian machine learning to identify key factors. The second stage applied a multilevel Bayesian regression to evaluate the state-level effects on stroke prevalence of these key factors.

We first used an iterative approach to remove the minimum number of highly correlated predictor variables as redundant predictors for stable model performance [20, 26, 27]. Alternative feature selection methods may be used for other study settings such as imaging data [28, 29] or classification problems [30, 31]. We then implemented a permutation-based variable selection algorithm, BART-Machine, developed in Bleich et al. to identify major determinants for the neighborhood-level prevalence of stroke [32]. BART-Machine uses the infrastructure of the most influential generative probabilistic machine learning model, Bayesian Additive Regression Trees (BART) [33]. BART has been shown to have better predictive performance than many supervised machine learning methods, including random forests, boosted models and neural nets, in a variety of study settings [33, 34]. Details of the BART model have been described elsewhere [19, 35]. BART-Machine uses the *variable inclusion proportions (VIPs)*, i.e., the proportion of times each variable is selected as a splitting rule divided by the total number of splitting rules in building the BART model, as the measure of variable importance. The variable selection procedure can be outlined as follows: i) Compute the VIP for each predictor from the BART model fitted to the observed data. ii) Permute the response variable and rebuild the model and compute the VIPs for all predictors, which we refer to as “null” VIPs. Repeat this process 100 times to create a null permutation distribution of the VIPs. iii) Include a predictor if its VIP from the observed data exceeds the 95% quantile of the distribution of the null VIPs.

The BART-Machine variable selection procedure can be implemented using the R package bartMachine. This permutation-based variable selection approach does not require any additional assumptions beyond those of the BART model. The sum-of-trees plus normal errors is a flexible assumption that performs well across a wide range of data settings, especially relative to methods that make stronger parametric demands [32]. A disadvantage of the BART permutation-based procedure is the computational cost associated with running BART models on multiple (e.g., 100) permutation sets. However, parallel computing on multiple cores can be used to speed up computation. Comparisons of the performance of BART-Machine versus other tree-based machine learning approaches, including random forests [36], [36] and boosting [37], have been described elsewhere and have shown that BART-Machine tends to identify the most parsimonious set of important predictors while maintaining high prediction accuracy [19].

To investigate the contribution of identified key determinants to the prevalence of stroke, we fitted a fully Bayesian multilevel linear regression model to all census tracts in the US. The model explicitly took into account that individual census tracts (first level) are clustered in the states (second level). This was accomplished by pooling information across clusters, which tends to improve estimates about each cluster. The improved estimation leads to several benefits, including improved estimates for repeat sampling caused by multiple observations arising from the same unit, improved estimates for imbalance in sampling, explicit modeling of variation among units or groups within the data, and avoiding averaging which can manufacture false confidence [38]. The multilevel model is specified as

$$ {y}_{ij}={\beta}_{0j}+{\beta}_{1j}{x}_{1 ij}+\dots +{\beta}_{kj}{x}_{kij}+{\epsilon}_{ij} $$

$$ {\boldsymbol{\beta}}_{\boldsymbol{j}}=\left[{\beta}_{0j},\dots, {\beta}_{kj}\right]^{\prime}\sim \mathrm{MVNormal}\left(\boldsymbol{\mu}, \boldsymbol{\Sigma} \right), $$

where *y*_*ij*_ is the prevalence of stroke for census tract *i* in state *j*, *x*_1*ij*_, …, *x*_*kij*_ are *k* key determinants for census tract *i* in state *j*, and ${\epsilon}_{ij}\sim N\left(0,{\sigma}_{\epsilon}^2\right)$ is the residual term assumed to normal. The parameters *β*_1*j*,_…, *β*_*kj*_, which respectively encode the effects of *k* key determinants, are allowed to vary across states and are assigned their own distributions. We have also allowed the intercept, *β*_0_, to vary across states in a similar manner. Let ***β***_***j***_ denote the vector of *β*_*j*_ ’s. We assume *β*_*j*_ ’s are realizations from a common, multivariate normal distribution, ***β***_***j***_ ∼ MVNormal(***μ***, **Σ**), where ***μ*** is a (*k* + 1) × 1 mean vector and **Σ** is a (*k* + 1) × (*k* + 1) covariance matrix.

For a fully Bayesian analysis, we place prior distributions on the parameters, for which we chose weakly-informative priors. We specify weakly informative priors as follows.

$$ \boldsymbol{\Sigma} =\boldsymbol{D}\left({\sigma}_{\beta}\right)\boldsymbol{RD}\left({\sigma}_{\beta}\right) $$

$$ \boldsymbol{R}\sim \mathrm{LKJCorr}(2) $$

$$ {\sigma}_l\sim \mathrm{Half}\ \mathrm{Cauchy}\left(0,1\right) $$

$$ {\sigma}_{\epsilon}\sim \mathrm{Half}\ \mathrm{Cauchy}\left(0,1\right) $$

$$ {\mu}_l\sim N\left(0,10\right),l=1,\dots, k+1, $$

where ***D***(*σ*_*β*_) is a diagonal matrix with each diagonal element *σ*_*l*_ representing the standard deviation of *β*_*lj*_, *l* = 1, …, *k* + 1, on which we specified a Half Cauchy (0,1) prior distribution, and ***R*** is the corresponding correlation matrix for which we assigned a LKJ-Correlation prior with a shape parameter of 2 as recommended in McElreath [19]. We also used Half Cauchy (0,1) prior for *σ*_*ϵ*_ and assumed a normal distribution for each mean of the *β*_*j*_ ’s, *μ*_*l*_, with large enough standard deviation to be noninformative.

We used R package brms to get full Bayesian statistical inference with Markov chain Monte Carlo (MCMC) sampling for our Bayesian multilevel model [39]. The brms package provides a flexible interface to fit Bayesian multilevel models using Stan, which is a state-of-the-art platform for statistical modeling and high-performance statistical computation [40]. We used Stan’s default no-U-turn sampler (NUTS), which is a highly efficient algorithm that avoids the random walk behavior and sensitivity to correlated parameters and allows faster convergence to high-dimensional target distributions [39, 41]. To ensure convergence of posterior distribution, we used four MCMC chains, each with 5000 iterations, of which the first 2000 iterations were warmup to calibrate the sampler, leading to a total of 12,000 posterior samples. The data analysis in this manuscript was conducted in 2020.

## Results

**Fig. 2:**
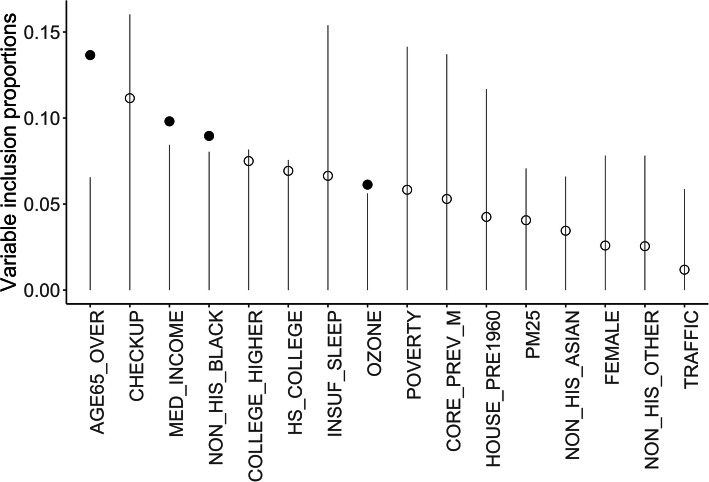
Visualization of the variable selection algorithm. The vertical lines are the threshold levels determined from the “null” distributions for Variable Inclusion Proportions computed from 100 permutated data. Variables passing this threshold are displayed as solid dots. Open dots correspond to variables that are not selected

We first identified and removed eight redundant variables. We then implemented BART-Machine to the remaining 16 variables and identified, for the prevalence of stroke at the neighborhood level, four most important predictors: the proportion of people who are older than 65, the proportion of non-Hispanic black, median household income and ambient ozone level. These four variables were selected as they all had proportion included above their corresponding thresholds, as shown in Fig. 2.

**Fig. 3:**
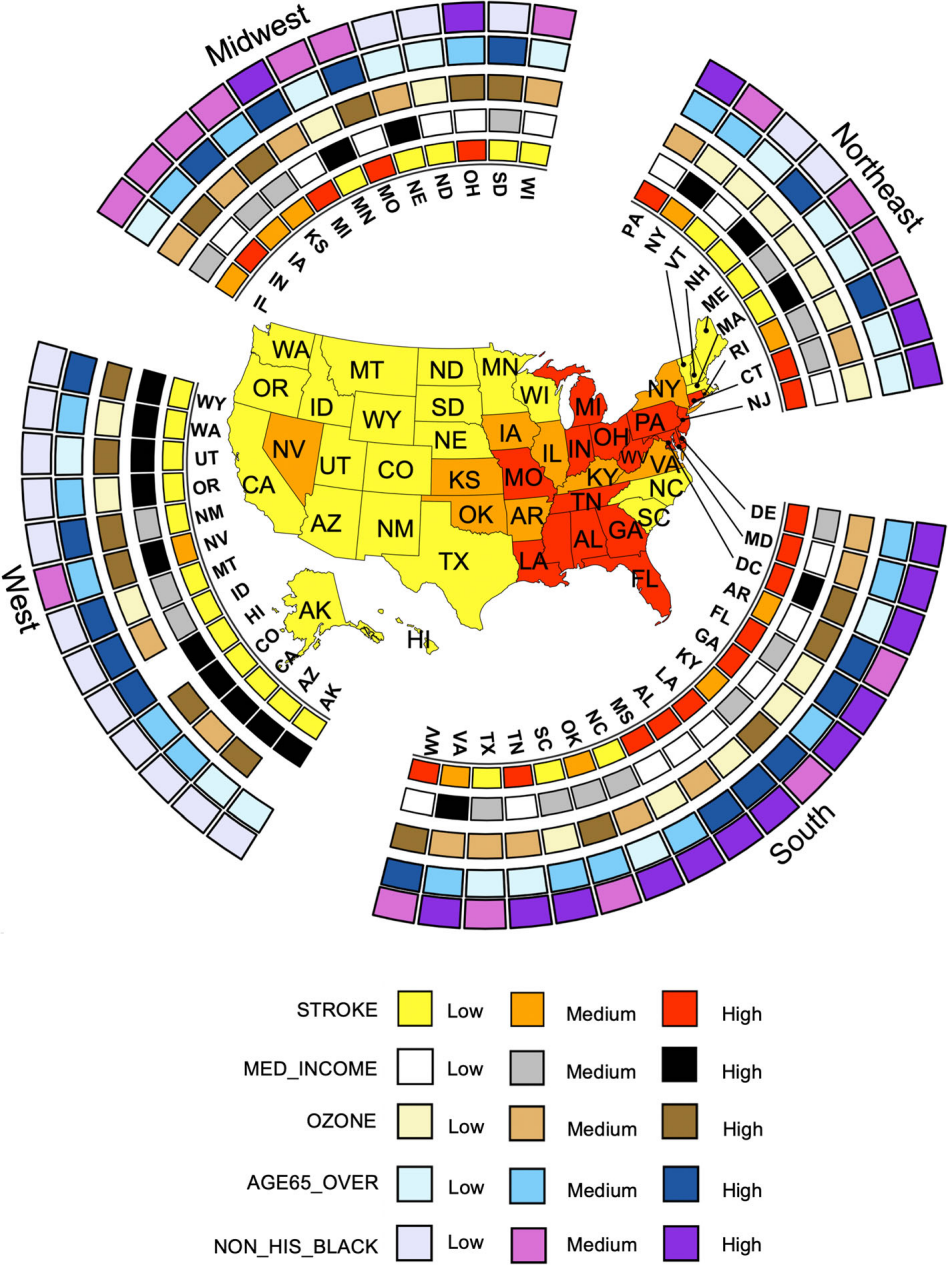
Ring map visualization of stroke prevalence and four major determinants for 50 states and the District of Columbia states. The median value of the measures of census tracts was used for each state. Low, Medium and High were categorized based on tertiles of the distribution of median values across the states. There were no ozone measures for Hawaii and Alaska. Ring map was created using the open source R software version 3.6.1. URL https://www.R-project.org/. The R codes are provided in the supplementary materials

We generated a ring map using the R software 3.6.2 in Fig. 3 to visualize the geographic variations in the prevalence of stroke and the four identified key determinants. The R codes can be found in the supplementary materials. Levels of these variables were categorized by tertiles. States with a high stroke prevalence were concentrated in the southern US. Ten out of 16 southern states had stroke prevalence ranked in the highest tertile. These ten states also tended to have low median household income and high proportion of non-Hispanic blacks. The northeast region appeared to have the lowest ozone level and youngest population, and the West has the highest median household income and the lowest prevalence of stroke. The proportion of older residents and non-Hispanic blacks is also higher overall in the South.

**Fig. 4:**
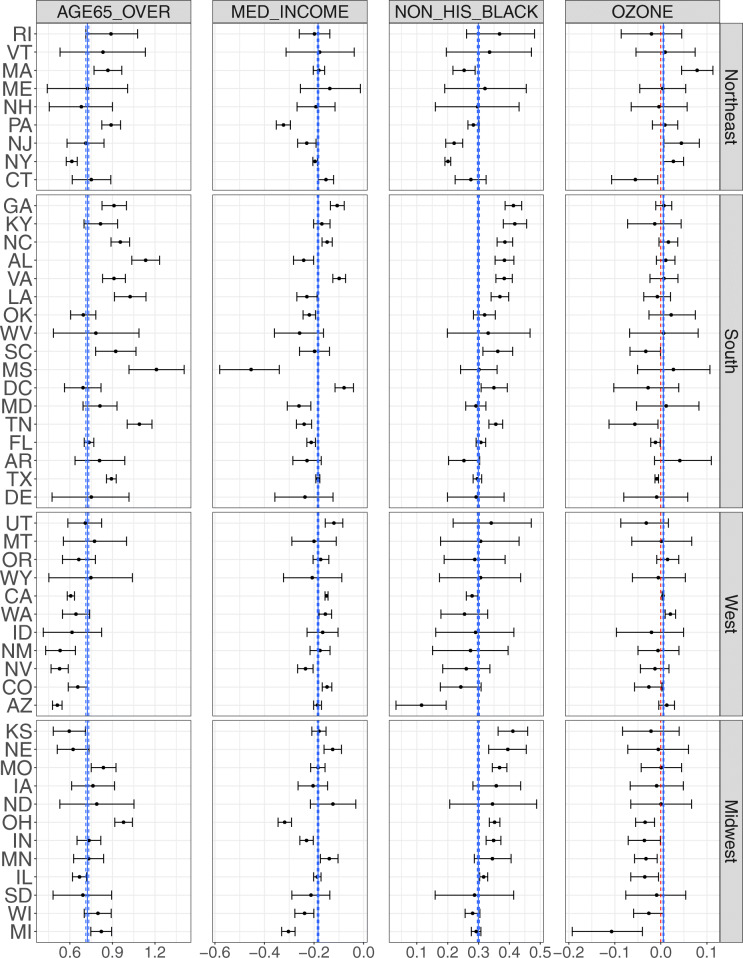
Posterior mean of the state-specific effects of four key neighborhood-level determinants (solid dots) and corresponding 95% credible intervals (error bars). Effect estimates represent average changes in percent of stroke per 10% increase in AGE65_OVER or NON_HIS_BLACK, and per $100,000 increase in MED_INCOME and per 10 ppb increase in OZONE. Results are shown for 49 states as there are no ozone measures for Hawaii and Alaska. The states are presented in four regions of the U.S. Blue lines represent the effects averaged across all states, ignoring variability in the states and yielding tightness of uncertainty intervals

Figure 4 summarizes the posterior distributions of the effects of the four key neighborhood characteristics obtained from our Bayesian multilevel model. First, overall, higher proportion of older inhabitants and larger share of non-Hispanic blacks were consistently associated with higher prevalence of stroke across the states. Medium household income was inversely associated with the prevalence of stroke. The ozone-stroke associations were found to be positive in 10 states, Connecticut, Florida, Illinois, Indiana, Michigan, Minnesota, Ohio, South Carolina, Tennessee and Texas. On the other hand, negative ozone-stroke associations were also found in California, Massachusetts, New Jersey, New York, Washington. Second, there appears to be substantial variation in the effect of each of four determinants across states with nonoverlapping credible intervals for some states. For example, the effect of median household income and advancing age were substantially stronger in Mississippi than in the District of Columbia (about 6 and 2 times larger effects, respectively, with nonoverlapping intervals.) The most pronounced effect of age structure was observed in Mississippi, and the effect of racial/ethnic composition was strongest in Georgia while smallest in Arizona.

## Discussion

The rise of machine learning techniques and algorithmic advances have enabled computer systems to carry out complex processes by learning from data, rather than following pre-programmed rules. The advent of the Big data era has stimulated novel approaches using machine learning techniques to generate relevant solutions -- faster and more accurately -- to impacting health. Machine learning has been used to predict healthcare outcomes including cost, utilization, and quality [42–45].

We leveraged a large-scale cardiovascular health dataset with information on unhealthy behaviors, prevention measures, sociodemographic status and environmental factors garnered from more than 20,000 census tracts in 500 US major cities. The key neighborhood-level determinants identified via the BART-Machine algorithm were in general in good agreement with known patient-level risk factors. Research from other studies also suggested a relationship between stroke and low neighborhood-level income [7, 9, 11–15, 19, 20], age [14, 15, 19, 20] and Hispanic blacks [8, 15–17, 19, 20]^.^

We employed a Bayesian multilevel modeling approach to attribute the geographic variability in the prevalence of stroke to major determinants while recognizing the hierarchical structure of the data set. Multilevel modeling improves estimates about each state by allowing partial pooling and explicitly modeling of variations among census tracts and states, which is particularly beneficial in the presence of highly unequal sample size – ranging from 11 to 5368 – across the states [38, 46, 47]. By explicitly modeling the variability in stroke prevalence across the states, our results provided a high resolution of how the neighborhood-level prevalence of stroke was attributed to key neighborhood characteristics across different states. Comparison of the blue lines representing the average effects with the state-specific posterior means and intervals in Fig. 4 suggests that ignoring the variability would lead to biased characterization of these associations, which can be directional (ozone effects) or of significant magnitude (age, income or non-Hispanic blacks).

Understanding these state-level variations is also important from the public health and policy perspectives, particularly for urban areas. In general, we found that neighborhoods with older and more non-Hispanic black populations tended to have a higher prevalence of stroke, while wealthier communities tended to have lower stroke prevalence. We also noted a stronger effect of income in poorer states (e.g. Mississippi, Ohio) than in affluent states (e.g. Virginia, District of Columbia). In addition to the region-specific effects estimated by our Bayesian multilevel modelling approach, our study was the first to shed lights on the association between stroke and ozone level both at the neighborhood level. We found that the effect of ozone on stroke prevalence at neighborhood-level is mixed, as it was found to be positively associated with stroke in less wealthy states while negatively associated with stroke in more affluent states. At individual-level, findings of the effect of ozone on stroke and stroke-related health outcomes have also been inconsistent. Montresor et al. and Wing et al. have demonstrated a negative association between average ozone levels and risk of strokes in South Carolina and Texas [48, 49]. Henrotin et al. found that there was a positive association between risk of ischemic stroke and daily ozone exposure, while Yu et al. suggested protective effects of ozone on incidence and outcomes of stroke [50–52]. Investigation into the link between ozone and cardiovascular health merits further research. These results are in line with those from patient-level studies and suggest that there is at present no general agreement about the effect of ozone on stroke and further research in this area is warranted.

Our study has important implications related to public health and policy. Identifying major neighborhood-level determinants allows in-depth investigation into the exposition of geographic variation in the prevalence of stroke by major neighborhood characteristics. The findings from our study can provide tailored area-based interventions to reduce the burden of cardiovascular disease. For example, interventions aimed at tackling disparities in the prevalence of stroke could focus on the older population structure in states like Mississippi and Alabama in the South region, and on ozone level in densely populated states like New York and Massachusetts. As the proportion of non-Hispanic blacks was shown to have the largest effect in Gregoria, Kansas and Kentucky, it may be critical for these states to address avoidable inequalities and to eliminate health and health care disparities [53].

There are several limitations to this study. First, the prevalence of stroke only reflects the proportion of population who are alive and have a history of stroke, therefore it may not accurately and completely reflect the incidence of stroke and severity of the disease, and is subject to survivor bias [54]. However, these measures offer the best evidence available for these specific areas and the small area estimation approach used by the CDC has been well validated [55]. Second, due to the nature of cross-sectional data and ecological design, we could not establish the causal association between predictors and stroke health outcomes. Our study results can potentially motivate future research on causality with patient-level longitudinal data [54, 56, 57]. Third, investigating the relationship between environmental risk factors such as ozone and cardiovascular health could be a worthwhile contribution. Finally, future efforts are needed to integrate neighborhood- and individual-level data and study how risk factors at the neighborhood level jointly impact stroke incidence and other health outcomes after stroke with individual characteristics, such as diet, education and social support. Ultimately it will be critical to incorporate these knowledge into interventions to improve stroke care at a population level.

## Conclusions

We used a large-scale neighborhood-level data on 500 cities in the US pooled from multiple sources, and implemented a two-stage approach to first identify the key determinants for stroke prevalence at the neighborhood level and then quantify the geographic variability in the effects of key determinants. This was the first study to contribute insights into the underlying effect mechanisms between neighborhood characteristics and stroke prevalence while taking into account the multilevel data structure, when both predictors and outcomes are measured at the neighborhood level. We used a state-of-the-art Bayesian machine learning technique in the first stage and the multilevel modelling approach for the second stage. We found that a higher proportion of older inhabitants and a larger share of non-Hispanic Blacks were associated with a higher prevalence of stroke across the states. Medium household income was inversely associated with the prevalence of stroke. Ozone was found to be positively associated with stroke prevalence in 10 states, while negatively associated with stroke in five states. There was substantial variation in the associations between these four key factors with stroke prevalence across the states, in both the direction and the magnitude. With the large sample size, wide-ranging data information and methodologically rigorous analyses, our study results improve our understanding of how neighborhood-level risk factors contribute to the neighborhood-level stroke prevalence in each state of the U.S., which can facilitate developing area-based stroke prevention strategies.

## Supplementary Information

**Additional file 1.** R code for Fig. 3.

## Data Availability

We used 3 datasets during the current study. CDC’s 500 Cities Project 2017 data release on 28,004 census tracts is publicly available on its website, https://chronicdata.cdc.gov/browse?category=500+Cities [16]. The 2011–2015 American Community Survey 5-Year Estimates is publicly available on the website, https://www.census.gov/data/developers/data-sets/acs-5year.html [17]. The EPA’s Environmental Justice Screening (EJSCREEN) database is also publicly available on the website, https://www.epa.gov/ejscreen/download-ejscreen-data [19].

## Abbreviations

CDC: Centers for Disease Control and Prevention
EPA: Environmental Protection Agency
EJSCREEN: Environmental justice screening
BRFSS: Behavioral Risk factor surveillance system
VIPs: Variable inclusion proportions
MCMC: Markov chain Monte Carlo
NUTS: No-U-turn sampler
BART: Bayesian additive regression trees
