# Supplementary material for: Identifying and assessing the impact of key neighborhood-level determinants on geographic variation in stroke: a machine learning and multilevel modeling approach
Source: BMC Public Health. 2020 Nov 7;20:1666. doi: 10.1186/s12889-020-09766-3 (PMC7648288; doi:10.1186/s12889-020-09766-3)
Supplement: Supplementary file 1 — Additional file 1. R code for Fig. 3. [file 12889_2020_9766_MOESM1_ESM.docx]

**R code for Figure 3.**

library(usmap)

library(tidyverse)

# Data cleaning -----------------------------------------------------------

map_data_STROKE <- read_csv("data/TractCdcAcsEJ_full.csv") %>%

filter(!(is.na(STROKE))) %>%

select(GeoLocation, STROKE,StateAbbr) %>%

separate(GeoLocation, c("lat", "lon"), sep = ",") %>%

# head %>% pull(lat)

mutate(lon = str_sub(lon, 2,-2),

lat = str_sub(lat, 2)) %>%

mutate(lon = as.numeric(lon),

lat = as.numeric(lat)) %>%

# mutate(lat = round(lat,3),

# lon = round(lon,3)) %>%

select(-StateAbbr) %>%

select(lon, lat, STROKE)

median_stroke_state <- read_csv("data/TractCdcAcsEJ_full.csv") %>%

filter(!(is.na(STROKE))) %>%

group_by(StateAbbr) %>%

summarise(median_stroke = median(STROKE))

median_stroke_state <- usmap::statepop %>%

select(fips, abbr, full) %>%

inner_join(median_stroke_state %>%

rename(abbr = StateAbbr)) %>%

mutate(median_stroke = cut_number(median_stroke, 3))

median_tmedinc_state <- read_csv("data/TractCdcAcsEJ_full.csv") %>%

filter(!(is.na(tmedinc))) %>%

group_by(StateAbbr) %>%

summarise(median_tmedinc = median(tmedinc)) %>%

mutate(median_tmedinc = median_tmedinc /1000)

median_tmedinc_state <- usmap::statepop %>%

select(fips, abbr, full) %>%

inner_join(median_tmedinc_state %>%

rename(abbr = StateAbbr)) %>%

mutate(median_tmedinc = cut_number(median_tmedinc, 3))

# mutate(median_tmedinc = case_when(median_tmedinc == "[30.9,43.4]" ~ "Low",

# median_tmedinc == "(43.4,50.5]" ~ "Medium",

# median_tmedinc == "(50.5,78.1]" ~ "High"))

levels(median_tmedinc_state$median_tmedinc) <- c("Low", "Medium", "High")

median_nhispbkp_state <- read_csv("data/TractCdcAcsEJ_full.csv") %>%

filter(!(is.na(nhispbkp))) %>%

group_by(StateAbbr) %>%

summarise(median_nhispbkp = median(nhispbkp)) %>%

mutate(median_nhispbkp = median_nhispbkp *100)

median_nhispbkp_state <- usmap::statepop %>%

select(fips, abbr, full) %>%

inner_join(median_nhispbkp_state %>%

rename(abbr = StateAbbr)) %>%

mutate(median_nhispbkp = cut_number(median_nhispbkp, 3))

levels(median_nhispbkp_state$median_nhispbkp) <- c("Low", "Medium", "High")

median_age65_overp_state <- read_csv("data/TractCdcAcsEJ_full.csv") %>%

filter(!(is.na(age65_overp))) %>%

group_by(StateAbbr) %>%

summarise(median_age65_overp = median(age65_overp)) %>%

mutate(median_age65_overp = median_age65_overp *100)

median_age65_overp_state <- usmap::statepop %>%

select(fips, abbr, full) %>%

inner_join(median_age65_overp_state %>%

rename(abbr = StateAbbr)) %>%

mutate(median_age65_overp = cut_number(median_age65_overp, 3))

levels(median_age65_overp_state$median_age65_overp) <- c("Low", "Medium", "High")

median_tractOZONE_state <- read_csv("data/TractCdcAcsEJ_full.csv") %>%

filter(!(is.na(tractOZONE))) %>%

group_by(StateAbbr) %>%

summarise(median_tractOZONE = median(tractOZONE))

median_tractOZONE_state <- usmap::statepop %>%

select(fips, abbr, full) %>%

full_join(median_tractOZONE_state %>%

rename(abbr = StateAbbr)) %>%

mutate(median_tractOZONE = cut_number(median_tractOZONE, 3))

levels(median_tractOZONE_state$median_tractOZONE) <- c("Low", "Medium", "High")

median_tractOZONE_state %>%

full_join(median_age65_overp_state) %>%

filter(is.na(median_tractOZONE))

# stroke circle -----------------------------------------------------------

median_stroke_state_circle_data <- median_stroke_state %>%

# filter(!(full %in% c("Alaska", "Hawaii"))) %>%

mutate(group = case_when(full %in% c("Maine", "Vermont", "New Hampshire", "Massachusetts", "Connecticut", "Rhode Island", "New York", "New Jersey","Pennsylvania") ~ "Northeast",

full %in% c("Ohio", "Illinois", "Indiana", "Michigan", "Wisconsin", "Minnesota", "Iowa", "Missouri", "North Dakota", "South Dakota", "Kansas","Nebraska") ~ "Midwest",

full %in% c("Delaware", "Maryland", "District of Columbia", "West Virginia", "Virginia", "Kentucky", "Tennessee", "North Carolina", "South Carolina", "Georgia", "Florida", "Alabama", "Mississippi", "Louisiana", "Oklahoma", "Arkansas", "Texas") ~ "South",

full %in% c("Washington", "Oregon", "California", "Nevada", "New Mexico", "Arizona", "Idaho", "Utah", "Colorado", "Montana", "Wyoming", "Alaska", "Hawaii") ~ "West"),

group = factor(group, levels = c("Northeast", "South", "West", "Midwest"))) %>%

arrange(group) %>%

select(-fips,-full) %>%

mutate(height = 1)

median_stroke_state_circle_seq <- median_stroke_state_circle_data %>% pull(1)

empty_bar <- 3

to_add <- data.frame( matrix(NA, empty_bar*nlevels(median_stroke_state_circle_data$group), ncol(median_stroke_state_circle_data)) )

colnames(to_add) <- colnames(median_stroke_state_circle_data)

to_add$group <- rep(levels(median_stroke_state_circle_data$group), each=empty_bar)

median_stroke_state_circle_data <- rbind(median_stroke_state_circle_data, to_add)

median_stroke_state_circle_data <- median_stroke_state_circle_data %>%

mutate(abbr = factor(abbr, levels = c("PA", "NY", "VT", "NH", "ME", "MA", "RI", "CT", "NJ", NA, NA, NA, "DE", "MD","DC", "AR", "FL","GA", "KY", "LA", "AL", "MS", "NC", "OK", "SC", "TN", "TX", "VA", "WV", NA, NA, NA, "AK", "AZ", "CA","CO","HI", "ID", "MT", "NV", "NM", "OR", "UT", "WA", "WY", NA, NA, NA, "IL", "IN", "IA", "KS", "MI","MN", "MO", "NE", "ND", "OH", "SD", "WI", NA, NA, NA)), exclude = NULL) %>%

arrange(group, abbr)

median_stroke_state_circle_data$id <- seq(1, nrow(median_stroke_state_circle_data))

# Get the name and the y position of each label

label_data <- median_stroke_state_circle_data

number_of_bar <- nrow(label_data)

angle <- 90 - 360 * (label_data$id+3.5) /number_of_bar # I substract 0.5 because the letter must have the angle of the center of the bars. Not extreme right(1) or extreme left (0)

label_data$hjust <- ifelse( angle < -90, 1, 0)

label_data$angle <- ifelse(angle < -90, angle+180, angle)

label_data$height <- -1.8

base_data <- median_stroke_state_circle_data %>%

group_by(group) %>%

summarize(start=min(id), end=max(id) - empty_bar) %>%

rowwise() %>%

mutate(title=mean(c(start, end))) %>%

mutate(start = start -0.5, end = end +0.5)

ggplot(median_stroke_state_circle_data, aes(x=as.factor(id), y=height, fill=median_stroke)) + # Note that id is a factor. If x is numeric, there is some space between the first bar

geom_bar(aes(x=as.factor(id), y=height, fill=median_stroke), stat="identity",color = "black") +

theme_minimal() +

ylim(-15,1) +

theme(

legend.position = "none",

axis.text = element_blank(),

axis.title = element_blank(),

panel.grid = element_blank(),

plot.margin = unit(rep(-1,4), "cm")

) +

coord_polar(start = 0.5) +

geom_text(data=label_data, aes(x=id, y=height, label=abbr, hjust=hjust), color="black", fontface="bold",alpha=1, size=4.5, angle= label_data$angle, inherit.aes = FALSE ) +

# # Add base line information

geom_segment(data=base_data, aes(x = start, y = -0.2, xend = end, yend = -0.2), colour = "black", alpha=0.8, size=0.6 , inherit.aes = FALSE ) +

# geom_text(data=base_data, aes(x = title, y = -0.3, label=group), hjust=c(1,1,0,0), colour = "black", alpha=0.8, size=4.5, fontface="bold", inherit.aes = FALSE)+

# scale_fill_gradientn(colours=rev(heat.colors(10)),na.value="grey90",

# guide = guide_colourbar(barwidth = 25, barheight = 0.4,

# #put legend title on top of legend

# title.position = "top"))

scale_fill_manual(values = c("#FFFB36", "#FEA400", "#FE2F00"))

ggsave("plot/circle.png",width = 7, height = 7, bg = "transparent")

# tmedinc circle ----------------------------------------------------------

median_tmedinc_state_circle_data <- median_tmedinc_state %>%

# filter(!(full %in% c("Alaska", "Hawaii"))) %>%

mutate(group = case_when(full %in% c("Maine", "Vermont", "New Hampshire", "Massachusetts", "Connecticut", "Rhode Island", "New York", "New Jersey","Pennsylvania") ~ "Northeast",

full %in% c("Ohio", "Illinois", "Indiana", "Michigan", "Wisconsin", "Minnesota", "Iowa", "Missouri", "North Dakota", "South Dakota", "Kansas","Nebraska") ~ "Midwest",

full %in% c("Delaware", "Maryland", "District of Columbia", "West Virginia", "Virginia", "Kentucky", "Tennessee", "North Carolina", "South Carolina", "Georgia", "Florida", "Alabama", "Mississippi", "Louisiana", "Oklahoma", "Arkansas", "Texas") ~ "South",

full %in% c("Washington", "Oregon", "California", "Nevada", "New Mexico", "Arizona", "Idaho", "Utah", "Colorado", "Montana", "Wyoming", "Alaska", "Hawaii") ~ "West"),

group = factor(group, levels = c("Northeast", "South", "West", "Midwest"))) %>%

arrange(group) %>%

select(-fips,-full) %>%

mutate(height = 1)

empty_bar <- 3

to_add <- data.frame( matrix(NA, empty_bar*nlevels(median_tmedinc_state_circle_data$group), ncol(median_tmedinc_state_circle_data)) )

colnames(to_add) <- colnames(median_tmedinc_state_circle_data)

to_add$group <- rep(levels(median_tmedinc_state_circle_data$group), each=empty_bar)

median_tmedinc_state_circle_data <- rbind(median_tmedinc_state_circle_data, to_add)

median_tmedinc_state_circle_data <- median_tmedinc_state_circle_data %>%

mutate(abbr = factor(abbr, levels = c("PA", "NY", "VT", "NH", "ME", "MA", "RI", "CT", "NJ", NA, NA, NA, "DE", "MD","DC", "AR", "FL","GA", "KY", "LA", "AL", "MS", "NC", "OK", "SC", "TN", "TX", "VA", "WV", NA, NA, NA, "AK", "AZ", "CA","CO","HI", "ID", "MT", "NV", "NM", "OR", "UT", "WA", "WY", NA, NA, NA, "IL", "IN", "IA", "KS", "MI","MN", "MO", "NE", "ND", "OH", "SD", "WI", NA, NA, NA)), exclude = NULL) %>%

arrange(group, abbr)

median_tmedinc_state_circle_data$id <- seq(1, nrow(median_tmedinc_state_circle_data))

# Get the name and the y position of each label

label_data <- median_tmedinc_state_circle_data

number_of_bar <- nrow(label_data)

angle <- 90 - 360 * (label_data$id+3.5) /number_of_bar # I substract 0.5 because the letter must have the angle of the center of the bars. Not extreme right(1) or extreme left (0)

label_data$hjust <- ifelse( angle < -90, 1, 0)

label_data$angle <- ifelse(angle < -90, angle+180, angle)

label_data$height <- -1.5

base_data <- median_tmedinc_state_circle_data %>%

group_by(group) %>%

summarize(start=min(id), end=max(id) - empty_bar) %>%

rowwise() %>%

mutate(title=mean(c(start, end))) %>%

mutate(start = start -0.5, end = end +0.5)

ggplot(median_tmedinc_state_circle_data, aes(x=as.factor(id), y=height, fill=median_tmedinc)) + # Note that id is a factor. If x is numeric, there is some space between the first bar

geom_bar(aes(x=as.factor(id), y=height, fill=median_tmedinc), stat="identity",color = "black") +

theme_minimal() +

ylim(-15,1) +

labs(fill = "MED_INCOME")+

guides(fill = guide_legend(nrow = 1))+

theme(

legend.position = "none",

axis.text = element_blank(),

axis.title = element_blank(),

panel.grid = element_blank(),

plot.margin = unit(rep(-1,4), "cm")

) +

coord_polar(start = 0.5) +

scale_fill_manual(values = c("white", "grey", "black"))

# geom_text(data=label_data, aes(x=id, y=height, label=abbr, hjust=hjust), color="black", fontface="bold",alpha=0.6, size=4.5, angle= label_data$angle, inherit.aes = FALSE ) +

#

# # Add base line information

# geom_segment(data=base_data, aes(x = start, y = -0.2, xend = end, yend = -0.2), colour = "black", alpha=0.8, size=0.6 , inherit.aes = FALSE ) +

# geom_text(data=base_data, aes(x = title, y = -0.3, label=group), hjust=c(1,1,0,0), colour = "black", alpha=0.8, size=4.5, fontface="bold", inherit.aes = FALSE)+

# scale_fill_gradientn(colours=c("white", "black"),na.value="grey90",

# guide = guide_colourbar(barwidth = 25, barheight = 0.4,

# #put legend title on top of legend

# title.position = "top"))

ggsave("plot/circle_tmedinc.png",width = 7, height = 7, bg = "transparent")

# age65_overp circle -------------------------------------------------------

median_age65_overp_state_circle_data <- median_age65_overp_state %>%

# filter(!(full %in% c("Alaska", "Hawaii"))) %>%

mutate(group = case_when(full %in% c("Maine", "Vermont", "New Hampshire", "Massachusetts", "Connecticut", "Rhode Island", "New York", "New Jersey","Pennsylvania") ~ "Northeast",

full %in% c("Ohio", "Illinois", "Indiana", "Michigan", "Wisconsin", "Minnesota", "Iowa", "Missouri", "North Dakota", "South Dakota", "Kansas","Nebraska") ~ "Midwest",

full %in% c("Delaware", "Maryland", "District of Columbia", "West Virginia", "Virginia", "Kentucky", "Tennessee", "North Carolina", "South Carolina", "Georgia", "Florida", "Alabama", "Mississippi", "Louisiana", "Oklahoma", "Arkansas", "Texas") ~ "South",

full %in% c("Washington", "Oregon", "California", "Nevada", "New Mexico", "Arizona", "Idaho", "Utah", "Colorado", "Montana", "Wyoming", "Alaska", "Hawaii") ~ "West"),

group = factor(group, levels = c("Northeast", "South", "West", "Midwest"))) %>%

arrange(group) %>%

select(-fips,-full) %>%

mutate(height = 1)

empty_bar <- 3

to_add <- data.frame( matrix(NA, empty_bar*nlevels(median_age65_overp_state_circle_data$group), ncol(median_age65_overp_state_circle_data)) )

colnames(to_add) <- colnames(median_age65_overp_state_circle_data)

to_add$group <- rep(levels(median_age65_overp_state_circle_data$group), each=empty_bar)

median_age65_overp_state_circle_data <- rbind(median_age65_overp_state_circle_data, to_add)

median_age65_overp_state_circle_data <- median_age65_overp_state_circle_data %>%

mutate(abbr = factor(abbr, levels = c("PA", "NY", "VT", "NH", "ME", "MA", "RI", "CT", "NJ", NA, NA, NA, "DE", "MD","DC", "AR", "FL","GA", "KY", "LA", "AL", "MS", "NC", "OK", "SC", "TN", "TX", "VA", "WV", NA, NA, NA, "AK", "AZ", "CA","CO","HI", "ID", "MT", "NV", "NM", "OR", "UT", "WA", "WY", NA, NA, NA, "IL", "IN", "IA", "KS", "MI","MN", "MO", "NE", "ND", "OH", "SD", "WI", NA, NA, NA)), exclude = NULL) %>%

arrange(group, abbr)

median_age65_overp_state_circle_data$id <- seq(1, nrow(median_age65_overp_state_circle_data))

# Get the name and the y position of each label

label_data <- median_age65_overp_state_circle_data

number_of_bar <- nrow(label_data)

angle <- 90 - 360 * (label_data$id+3.5) /number_of_bar # I substract 0.5 because the letter must have the angle of the center of the bars. Not extreme right(1) or extreme left (0)

label_data$hjust <- ifelse( angle < -90, 1, 0)

label_data$angle <- ifelse(angle < -90, angle+180, angle)

label_data$height <- -1.5

base_data <- median_age65_overp_state_circle_data %>%

group_by(group) %>%

summarize(start=min(id), end=max(id) - empty_bar) %>%

rowwise() %>%

mutate(title=mean(c(start, end))) %>%

mutate(start = start -0.5, end = end +0.5)

ggplot(median_age65_overp_state_circle_data, aes(x=as.factor(id), y=height, fill=median_age65_overp)) + # Note that id is a factor. If x is numeric, there is some space between the first bar

geom_bar(aes(x=as.factor(id), y=height, fill=median_age65_overp), stat="identity",color = "black") +

theme_minimal() +

# labs()

ylim(-15,1) +

guides(fill = guide_legend(nrow = 1))+

theme(

legend.position = "none",

axis.text = element_blank(),

axis.title = element_blank(),

panel.grid = element_blank(),

plot.margin = unit(rep(-1,4), "cm")

) +

coord_polar(start = 0.5) +

scale_fill_manual(values = c("#DBF5FC", "#7ECEFA", "#1F57A3"))

# geom_text(data=label_data, aes(x=id, y=height, label=abbr, hjust=hjust), color="black", fontface="bold",alpha=0.6, size=4.5, angle= label_data$angle, inherit.aes = FALSE ) +

#

# # Add base line information

# geom_segment(data=base_data, aes(x = start, y = -0.2, xend = end, yend = -0.2), colour = "black", alpha=0.8, size=0.6 , inherit.aes = FALSE ) +

# geom_text(data=base_data, aes(x = title, y = -0.3, label=group), hjust=c(1,1,0,0), colour = "black", alpha=0.8, size=4.5, fontface="bold", inherit.aes = FALSE)+

# scale_fill_gradientn(na.value="grey90",

# guide = guide_colourbar(barwidth = 25, barheight = 0.4,

# #put legend title on top of legend

# title.position = "top"))

ggsave("plot/circle_age65_overp.png",width = 7, height = 7, bg = "transparent")

# nhispbkp circle -------------------------------------------------------

median_nhispbkp_state_circle_data <- median_nhispbkp_state %>%

# filter(!(full %in% c("Alaska", "Hawaii"))) %>%

mutate(group = case_when(full %in% c("Maine", "Vermont", "New Hampshire", "Massachusetts", "Connecticut", "Rhode Island", "New York", "New Jersey","Pennsylvania") ~ "Northeast",

full %in% c("Ohio", "Illinois", "Indiana", "Michigan", "Wisconsin", "Minnesota", "Iowa", "Missouri", "North Dakota", "South Dakota", "Kansas","Nebraska") ~ "Midwest",

full %in% c("Delaware", "Maryland", "District of Columbia", "West Virginia", "Virginia", "Kentucky", "Tennessee", "North Carolina", "South Carolina", "Georgia", "Florida", "Alabama", "Mississippi", "Louisiana", "Oklahoma", "Arkansas", "Texas") ~ "South",

full %in% c("Washington", "Oregon", "California", "Nevada", "New Mexico", "Arizona", "Idaho", "Utah", "Colorado", "Montana", "Wyoming", "Alaska", "Hawaii") ~ "West"),

group = factor(group, levels = c("Northeast", "South", "West", "Midwest"))) %>%

arrange(group) %>%

select(-fips,-full) %>%

mutate(height = 1)

empty_bar <- 3

to_add <- data.frame( matrix(NA, empty_bar*nlevels(median_nhispbkp_state_circle_data$group), ncol(median_nhispbkp_state_circle_data)) )

colnames(to_add) <- colnames(median_nhispbkp_state_circle_data)

to_add$group <- rep(levels(median_nhispbkp_state_circle_data$group), each=empty_bar)

median_nhispbkp_state_circle_data <- rbind(median_nhispbkp_state_circle_data, to_add)

median_nhispbkp_state_circle_data <- median_nhispbkp_state_circle_data %>%

mutate(abbr = factor(abbr, levels = c("PA", "NY", "VT", "NH", "ME", "MA", "RI", "CT", "NJ", NA, NA, NA, "DE", "MD","DC", "AR", "FL","GA", "KY", "LA", "AL", "MS", "NC", "OK", "SC", "TN", "TX", "VA", "WV", NA, NA, NA, "AK", "AZ", "CA","CO","HI", "ID", "MT", "NV", "NM", "OR", "UT", "WA", "WY", NA, NA, NA, "IL", "IN", "IA", "KS", "MI","MN", "MO", "NE", "ND", "OH", "SD", "WI", NA, NA, NA)), exclude = NULL) %>%

arrange(group, abbr)

median_nhispbkp_state_circle_data$id <- seq(1, nrow(median_nhispbkp_state_circle_data))

# Get the name and the y position of each label

label_data <- median_nhispbkp_state_circle_data

number_of_bar <- nrow(label_data)

angle <- 90 - 360 * (label_data$id+3.5) /number_of_bar # I substract 0.5 because the letter must have the angle of the center of the bars. Not extreme right(1) or extreme left (0)

label_data$hjust <- ifelse( angle < -90, 1, 0)

label_data$angle <- ifelse(angle < -90, angle+180, angle)

label_data$height <- -1.5

base_data <- median_nhispbkp_state_circle_data %>%

group_by(group) %>%

summarize(start=min(id), end=max(id) - empty_bar) %>%

rowwise() %>%

mutate(title=mean(c(start, end))) %>%

mutate(start = start -0.5, end = end +0.5)

ggplot(median_nhispbkp_state_circle_data, aes(x=as.factor(id), y=height, fill=median_nhispbkp)) + # Note that id is a factor. If x is numeric, there is some space between the first bar

geom_bar(aes(x=as.factor(id), y=height, fill=median_nhispbkp), stat="identity",color = "black") +

theme_minimal() +

ylim(-15,1) +

labs(fill = "NON_HIS_BLACK \n AGE65_OVER")+

guides(fill = guide_legend(nrow = 1))+

theme(

legend.position = "none",

axis.text = element_blank(),

axis.title = element_blank(),

panel.grid = element_blank(),

plot.margin = unit(rep(-1,4), "cm")

) +

coord_polar(start = 0.5) +

scale_fill_manual(values = c("#E6E6FA", "#DA70D6", "#8A2BE2"))

# geom_text(data=label_data, aes(x=id, y=height, label=abbr, hjust=hjust), color="black", fontface="bold",alpha=0.6, size=4.5, angle= label_data$angle, inherit.aes = FALSE ) +

#

# # Add base line information

# geom_segment(data=base_data, aes(x = start, y = -0.2, xend = end, yend = -0.2), colour = "black", alpha=0.8, size=0.6 , inherit.aes = FALSE ) +

# geom_text(data=base_data, aes(x = title, y = -0.3, label=group), hjust=c(1,1,0,0), colour = "black", alpha=0.8, size=4.5, fontface="bold", inherit.aes = FALSE)+

# scale_fill_gradientn(na.value="grey90",

# guide = guide_colourbar(barwidth = 25, barheight = 0.4,

# #put legend title on top of legend

# title.position = "top"))

scale_fill_gradient(limits = c(0,100))

ggsave("plot/circle_nhispbkp.png",width = 7, height = 7, bg = "transparent")

# tractOZONE circle -------------------------------------------------------

median_tractOZONE_state_circle_data <- median_tractOZONE_state %>%

# filter(!(full %in% c("Alaska", "Hawaii"))) %>%

mutate(group = case_when(full %in% c("Maine", "Vermont", "New Hampshire", "Massachusetts", "Connecticut", "Rhode Island", "New York", "New Jersey","Pennsylvania") ~ "Northeast",

full %in% c("Ohio", "Illinois", "Indiana", "Michigan", "Wisconsin", "Minnesota", "Iowa", "Missouri", "North Dakota", "South Dakota", "Kansas","Nebraska") ~ "Midwest",

full %in% c("Delaware", "Maryland", "District of Columbia", "West Virginia", "Virginia", "Kentucky", "Tennessee", "North Carolina", "South Carolina", "Georgia", "Florida", "Alabama", "Mississippi", "Louisiana", "Oklahoma", "Arkansas", "Texas") ~ "South",

full %in% c("Washington", "Oregon", "California", "Nevada", "New Mexico", "Arizona", "Idaho", "Utah", "Colorado", "Montana", "Wyoming", "Alaska", "Hawaii") ~ "West"),

group = factor(group, levels = c("Northeast", "South", "West", "Midwest"))) %>%

arrange(group) %>%

select(-fips,-full) %>%

mutate(height = 1)

empty_bar <- 3

to_add <- data.frame( matrix(NA, empty_bar*nlevels(median_tractOZONE_state_circle_data$group), ncol(median_tractOZONE_state_circle_data)) )

colnames(to_add) <- colnames(median_tractOZONE_state_circle_data)

to_add$group <- rep(levels(median_tractOZONE_state_circle_data$group), each=empty_bar)

median_tractOZONE_state_circle_data <- rbind(median_tractOZONE_state_circle_data, to_add)

median_tractOZONE_state_circle_data <- median_tractOZONE_state_circle_data %>%

mutate(abbr = factor(abbr, levels = c("PA", "NY", "VT", "NH", "ME", "MA", "RI", "CT", "NJ", NA, NA, NA, "DE", "MD","DC", "AR", "FL","GA", "KY", "LA", "AL", "MS", "NC", "OK", "SC", "TN", "TX", "VA", "WV", NA, NA, NA, "AK", "AZ", "CA","CO","HI", "ID", "MT", "NV", "NM", "OR", "UT", "WA", "WY", NA, NA, NA, "IL", "IN", "IA", "KS", "MI","MN", "MO", "NE", "ND", "OH", "SD", "WI", NA, NA, NA)), exclude = NULL) %>%

arrange(group, abbr)

median_tractOZONE_state_circle_data$id <- seq(1, nrow(median_tractOZONE_state_circle_data))

# Get the name and the y position of each label

label_data <- median_tractOZONE_state_circle_data

number_of_bar <- nrow(label_data)

angle <- 90 - 360 * (label_data$id+3.5) /number_of_bar # I substract 0.5 because the letter must have the angle of the center of the bars. Not extreme right(1) or extreme left (0)

label_data$hjust <- ifelse( angle < -90, 1, 0)

label_data$angle <- ifelse(angle < -90, angle+180, angle)

label_data$height <- -1.5

base_data <- median_tractOZONE_state_circle_data %>%

group_by(group) %>%

summarize(start=min(id), end=max(id) - empty_bar) %>%

rowwise() %>%

mutate(title=mean(c(start, end))) %>%

mutate(start = start -0.5, end = end +0.5)

ggplot(median_tractOZONE_state_circle_data, aes(x=as.factor(id), y=height, fill=median_tractOZONE)) + # Note that id is a factor. If x is numeric, there is some space between the first bar

geom_bar(aes(x=as.factor(id), y=height, fill=median_tractOZONE), stat="identity",color = "black") +

theme_minimal() +

ylim(-15,1) +

labs(fill="OZONE")+

guides(fill = guide_legend(nrow = 1))+

theme(

legend.position = "none",

axis.text = element_blank(),

axis.title = element_blank(),

panel.grid = element_blank(),

plot.margin = unit(rep(-1,4), "cm")

) +

coord_polar(start = 0.5) +

scale_fill_manual(values = c("#F9F6C2", "#E3B36C", "#977332"))

# geom_text(data=label_data, aes(x=id, y=height, label=abbr, hjust=hjust), color="black", fontface="bold",alpha=0.6, size=4.5, angle= label_data$angle, inherit.aes = FALSE ) +

#

# # Add base line information

# geom_segment(data=base_data, aes(x = start, y = -0.2, xend = end, yend = -0.2), colour = "black", alpha=0.8, size=0.6 , inherit.aes = FALSE ) +

# geom_text(data=base_data, aes(x = title, y = -0.3, label=group), hjust=c(1,1,0,0), colour = "black", alpha=0.8, size=4.5, fontface="bold", inherit.aes = FALSE)+

# scale_fill_gradientn(na.value="grey90",

# guide = guide_colourbar(barwidth = 25, barheight = 0.4,

# #put legend title on top of legend

# title.position = "top"))

# scale_fill_gradient(limits = c(0,100))

ggsave("plot/circle_tractOZONE.png",width = 7, height = 7, bg = "transparent")
